# The British Hospitals Association Meeting

**Published:** 1912-07-06

**Authors:** 


### THE NATIONAL INSURANCE ACT AND HOSPITALS IN SCOTLAND.

### The British Hospitals Association Meeting.

### THE MEDICAL COMMISSIONER FOR SCOTLAND CROSS-EXAMINED.

A meeting of the Scottish members of the British Hospitals Association and Representatives from the Boards of Management of Scottish Hospitals was held in the Merchants' Hall, George Square, Glasgow, on the afternoon of Friday, June 28, 1912, for the purpose of considering what attitude should be taken by voluntary hospitals in regard to the treatment of insured persons under the National Insurance Act, 1911, suffering from tuberculosis, and also for considering what arrangement hospital managers proposed to make for the medical treatment of their nursing and domestic staff when the Insurance Act came into force, and for transacting any other competent business.

On the motion of Sir Robert Cranston, Colonel J. A. Roxburgh was called upon to preside.

Representatives were present from the Royal Infirmary, Edinburgh; the Western and Victoria Infirmaries, Glasgow; County Hospital, Ayr; Greenock Combination Hospital; Greenock Infirmary; Kilmarnock Infirmary; Glasgow Parish Council; Corporation of Edinburgh; Corporation of Glasgow; Aberdeen Royal Infirmary; Royal Hospital for Sick Children, Glasgow; Lock Hospital, Glasgow; Royal Infirmary, Dundee; the Dumfries and Galloway Royal Infirmary; Cancer Hospital, Glasgow; Eye Infirmary, Glasgow; Bridge of Weir Sanatorium; Royal Alexandra Infirmary, Paisley, etc.

Dr. D. J. Mackintosh, M.V.O., Honorary Secretary, intimated letters of apology from Sir Matthew Arthur, Mr. J. D. Hedderwick, D.L., Professor Bryce, Rev. J. Montgomerie Campbell, Mr. W. G. Black, LL.D., and others.

The Chairman read the following letter received from Dr. Macfarlane, the Honorary Secretary of the Glasgow and West of Scotland Branch of the British Medical Association :—

Refusal of Free Hospital Service to Insured Patients.

"Dear Sir,—The following resolution was carried at a meeting of the hospital staffs of the various general and special hospitals of the city (Glasgow), and I was instructed to forward a copy to you as Secretary of the British Hospitals Association. The motion is as follows :

"'That we, the visiting staffs of the various hospitals of this city, will not give any professional service to any insured person except in cases of urgency, whether indoor or outdoor relief is desired, unless terms are settled to the satisfaction of the profession.

"'Before signing Pledge I. we feel it is due to our directors to have a conference with them.'"

### The Chairman's Speech.

This is a matter, said the Chairman, that must be taken up by the managers of the separate hospitals, and is not one on which we are called to pronounce an opinion to-day. We are met in connection with the British Hospitals Association, as you know. The objects of that Association are to bring together those who are interested in British hospitals and as far as possible to try to get them to work together for the benefit of the hospitals as a whole. The business before us to-day is in connection with the Insurance Act, and there are various questions which have arisen which we think it would be well that as managers of voluntary hospitals throughout the country we should have an opportunity of discussing. I am very glad to say that we have got with us Dr. M'Vail, the Medical Commissioner for Scotland, who will be glad to answer any questions that may be put to him. I would just like to say that although this is a meeting of the British Hospitals Association, we have asked hospitals which are not represented on that Association to send delegates to this meeting, as we wish to get as representative an opinion on these points as possible.

The First Question for the Members.

I am asked to say that hospitals and infirmaries may be admitted to the Association on payment of a subscription of one guinea for hospitals with 100 beds and upwards with a right to nominate two members, and 10s. 6d. for hospitals of not more than 100 beds with the right to nominate one member. I am sure you will agree with me that the hospitals should have the opportunity which this Association gives them to confer together on matters which are likely to affect them in the future. To-day certain matters may be discussed, but it must be understood that we, as representatives, are not empowered to give any ruling or to come under any obligation till we have first reported to our respective Boards of Management. We are here to get as much information as we can and go back to our Boards and give a report of this meeting. There are, I think, certain points on which no doubt we will receive valuable information from Dr. M'Vail and great assistance in getting our Boards to come to a satisfactory decision. The first question we are called upon to discuss is to consider what attitude should be taken by voluntary hospitals in regard to the treatment of insured persons under the National Insurance Act, 1911, suffering from tuberculosis. There seems to have got into the minds of the public an idea that the sanatorium treatment provided for under the Act is entirely for pulmonary tuberculosis, but the Act makes reference to tuberculosis as a whole, and most of us know that there is anything from 8 to 10 per cent. at present in our general hospitals who are tuberculous patients, and one of the things that we would like to know, if Dr. M'Vail can tell us, is whether we who are managers of general hospitals are entitled to any benefit from the Act if we treat tuberculous patients, even although they are not suffering from phthisis, and whether we are entitled to any benefit for those patients we are already treating. Perhaps the simplest way to get on would be to ask Dr. M'Vail to give us the information.

Tuberculous Patients in Scotch Hospitals.

Dr. M'Vail : Mr. Chairman, ladies and gentlemen, I am very glad to be here to accede to the request which Dr. Mackintosh sent to me a day or two ago to attend this meeting; I shall be very glad to give you as much information as I can. The first question asked by the Chairman is with regard to cases of tuberculosis other than phthisis as to payment to voluntary hospitals for treating these cases. As he has pointed out, sanatorium benefit in the sense of the Act is not confined to the use of sanatoria in the ordinary sense. Sanatorium benefit has a statutory meaning in the Act. It includes the treatment of all forms of tuberculosis, and, if it be so determined, of other diseases than tuberculosis no doubt more or less closely allied to it. It is not confined to tuberculosis of the lungs, and it may include tuberculosis of the bones, the glands and the skin—all other forms of tuberculosis; but everything depends upon cases being recommended for treatment by the Insurance Committees. It is the Insurance Committees who are responsible for the financial arrangements or payment of cases, and it will be in their discretion entirely as to what cases may be recommended for treatment.

### An Example from Glasgow.

There will be for the City of Glasgow one Insurance Committee composed, as required by the statute, of three-fifths of the representatives of insured persons, one-fifth appointed by the Town Council, and a fifth appointed otherwise, and it lies entirely with that Committee to recommend cases. As regards existing cases and cases at present in the hospitals, it is obvious that these have not been recommended by the Insurance Committees, because the Committees are not yet in existence, but when they come into existence their functions relate only to insured persons. That at once excludes all under sixteen years old and includes of course only persons belonging to the insured class as defined in the Act, and amongst these insured persons it lies with the Insurance Committee to determine in each case whether sanatorium benefit shall be provided for that case. Having determined that, it then remains with the Insurance Committee to decide what shall be the kind of treatment—not the treatment, but the kind of treatment—whether it shall be treatment in an institution or treatment outside of an institution. If in an institution, it may be in a sanatorium, if the case is suitable for it, or it may be in an hospital, but independently of that it may be domiciliary by a medical attendant, so that the answer is that as regards cases at present in hospital I do not see that they can be the subject of payment out of the Sanatorium Benefit Fund, and when the Insurance Committees are constituted it will lie with them to recommend or not to recommend the payment of cases such as that.

The Chairman: I understand the sanatorium benefit begins at once.

Dr. M'Vail: Yes.

The Chairman: How can it begin at once if there is no Insurance Committee?

Dr. M'Vail: The Insurance Committees will be formed at once. They are due to be formed in time for the beginning of the Act.

### The L.G.B. and the Voluntary Hospitals.

The Chairman: I have here a letter sent to various hospitals from the Local Government Board, Edinburgh, dated June 3, which I shall read:—

*National Insurance Act,* 1911.

As you are doubtless aware, sanatoria and other institutions in Scotland, in which insured persons under the above Act suffering from tuberculosis are received for treatment, must first be approved by this Board.

If it is proposed to arrange with Insurance Committees for the treatment of such persons in your institution, I shall be glad if you will inform me by return so that arrangements may be made for the necessary inspection of the institution by an officer of the Board.—I am, etc., (Signed) John T. Maxwell, Secretary.

The managers of the hospitals have not yet sent a reply, and the question that Dr. M'Vail has answered will enable us to see the position we are in, but really at present there is no need, I think, of us applying for inspection. Is not that so?

### Hospital Beds and Sanatoria Benefit.

Dr. M'Vail: That lies with the Local Government Board and not with the Insurance Commissioners. We had a meeting yesterday with the Local Government Board, and I learned at that meeting that there are altogether in Scotland about eighty-two institutions available for the treatment of tuberculosis throughout the country, but I think it would be well for the voluntary hospitals to consider whether they should not have the present arrangement brought before the Board so as to determine whether the beds in their hospitals would be available for sanatorium benefit.

Mr. Love (Greenock): Do these include all the voluntary hospitals?

Dr. M'Vail: They include all kinds of hospitals in Glasgow. I am quite sure that in Glasgow they will include institutions like the Western Infirmary, the Victoria Infirmary, the Royal Hospital for Sick Children, and the Glasgow Corporation Dispensaries, the Bellfield Dispensary, and the Glasgow Central Dispensary.

Mr. Motion: Does it include the Poorhouse Hospitals?

Dr. M'Vail: I would not expect so.

Mr. Motion: From the terms of the Act?

Dr. M'Vail: Quite.

The Chairman: It is just a question whether we can apply now or wait till the medical benefit under the Act is decided. Probably that is the better thing to do, but if anybody has any question to ask Dr. M'Vail on this branch of the subject, we will be very glad to have it now.

Dr. M'Vail: Sanatorium benefit is quite distinct from medical benefit. We all know that.

Mr. Love (Greenock): I should like to ask if that circular referred to from the Local Government Board has been sent out broadcast, because, as far as I am aware, it has not been sent to us.

### The Chairman's Resolution.

The Chairman: We are not aware to whom it has been sent, but several institutions have got it—the larger ones, at any rate. If nobody else has anything to say on this branch of the subject, I think our views might be concentrated in this resolution—"That in the opinion of this conference, it is desirable to delay giving any reply to the circular-letter of the Local Government Board in regard to the treatment of tuberculosis in voluntary hospitals until arrangements have been made with the medical profession regarding medical benefit under the Act." The principal reason for that is that if the voluntary hospitals are going to receive remuneration for treating tuberculous patients, more than likely the medical officers who will treat these patients will require remuneration also, and, as things are at present, we don't know the position, and it would be well, it seems to me, to delay doing anything in the way of replying to this circular, or at least only acknowledge it, till the medical benefit has been definitely arranged To keep this in order, I beg to move that resolution.

Dr. Ebenezer Duncan: I would take the opportunity of seconding this resolution, because I want to say a few words upon it. In the first place, this is an enormous question—this question of treating tuberculosis. You, sir, have stated, a somewhat indefinite number, that from 8 to 10 per cent. of the patients in our hospitals are suffering from tuberculosis, but I would put it very much higher than that. We have a large number of patients suffering from tuberculosis affecting all parts of the body, particularly the glands and the joints, and also the bones, and in every hospital we have a very large number not only of such surgical cases, but we have also a very large number of medical cases of tuberculosis, affecting all parts of the body. I know, as a matter of fact, that in every hospital in Glasgow we have at all times a certain number of cases of pulmonary tuberculosis. It is perfectly true that in the Western Infirmary, I understand, and in the Royal Infirmary, there is an attempt made to exclude as far as possible pulmonary tuberculosis, but, as a matter of fact and as a matter of experience, that disease comes into these hospitals under all kinds of names, and we are bound occasionally to find when we examine our patients that instead of having the disease which has been mentioned in the line of admission, these patients are suffering in a number of cases from pulmonary tuberculosis, and I know from my experience in examining students in the Western Infirmary and the Royal Infirmary that there never was any difficulty in finding a sufficient number of pulmonary cases of tuberculosis being treated in these hospitals for the examination of students upon that disease.

### Tuberculous Patients in Victoria Infirmary.

In the Victoria Infirmary, with which I am naturally familiar, as a former physician, we have always had pulmonary tuberculosis, and we have two rooms set apart for the treatment of pulmonary tuberculosis, but in addition to these cases that we treat separately, I know, as a matter of fact, that these cases are commonly treated in the wards, and necessarily treated in the wards, in connection with other diseases. We have not only medical diseases, but even surgical diseases which are associated with tubercle, and in a very large number of cases I have been called into consultation by surgeons who have put to me the question whether it was desirable to perform operations, because they found that patients had tuberculosis of the lungs, and I have seen many suffering from advanced tuberculosis of the lungs. It is an enormous question, and I question whether we should take up these insured patients. It is one of the most serious positions to put before the directors of voluntary hospitals. We could not possibly entertain any proposal which would mean that we should take into our hospitals any very large number of such cases. We are there as voluntary hospitals for the treatment of the poor, and we have an abundance of applicants of the class of the necessitous that we desire to treat in the voluntary hospitals without considering the question of taking in persons for payment under this Act. I think it will be necessary for the Government to provide treatment of that description elsewhere.

### Dr. Ebenezer Duncan's Views.

That is my own private opinion, and I would deprecate very much our hospitals being competitors for cases of that class under the Insurance Act. I think that is the position that we are bound to take up at the present moment—that we cannot see our way, at least at present, to do anything under the Insurance Act, and I have no doubt it will be very difficult for the Insurance Commissioners to get those committees that Dr. M'Vail speaks of constituted in the way that the Act desires them to be constituted, by representatives being on these bodies from the medical profession. The medical profession in Glasgow, at any rate in the large majority of the divisions, will refuse to have anything whatever to do with these medical committees unless reasonable payments to the medical profession are made by the Commissioners or by the Government. I have very great pleasure in seconding the resolution.

### An Amendment.

Mr. W. A. Tait (Edinburgh) read a letter which the Edinburgh Royal Infirmary have sent to the Local Government Board: "Referring to your letter of 3rd inst., already acknowledged, I am now instructed by the Managers of the Royal Infirmary to say that, in the meantime, they do not propose to arrange with the Insurance Committee for the treatment of insured persons under the above Act, suffering from tuberculosis." I would like to suggest that in the resolution which has been proposed and seconded, it might be better to delete the reference to the medical benefit, and probably the resolution would be simpler if it said, "In the opinion of this Conference, it is desirable to delay giving any reply to the circular letter of the Local Government Board in regard to the treatment of tuberculosis in voluntary hospitals till the question be further considered."

Mr. Tait's suggestion was unanimously adopted.

### Questions for the L.G.B.

Mr. R. F. Barclay: Speaking as the Hon. Secretary of the Royal Hospital for Sick Children, I should like to submit the following correspondence which I had with the Local Government Board. I wrote the following letter on June 6, 1912, to the Secretary, Local Government Board, Edinburgh.

*National Insurance Act*, 1911.

I have your two circular-letters of 3rd inst. relative to this hospital and dispensary or out-patient department in connection therewith. To enable my directors to deal with the matter, I shall be obliged by your informing me on the following points:—

1. Does the term "tuberculosis" include not only phthisis but every form of tubercular disease in any part of the body?
2. Does the term "received for treatment" refer only to in-patients resident in an institution and treated there, or to out-patients resident in their own homes who received treatment at an out-patient department or dispensary of an hospital? From the fact that one of your letters refers to our dispensary, I presume the term refers to both kinds of treatment.
3. What grant or benefit will the institution carrying on such treatment be entitled to or receive under the Act, and what supervision or control will be claimed or exercised by the Local Government Board or the Insurance Commissioners or other authorities under the Act?
4. This hospital does not treat any "insured persons," but only children under twelve years of age, that is to say, I presume, "dependents" within the meaning of the Act. The Act is by no means clear as to the position of dependents, and I shall be obliged by your informing me on this matter, which is most important from the point of view of this hospital. What are the position and rights of dependents; what benefits are they to get; what is to be contributed for their treatment; from whom is payment to be received, etc.? Presumably the Commissioners have issued regulations on this matter. Please let me have a copy.

I may say that not only at the hospital and dispensary, but also at the country branch of this hospital, many of the patients treated suffer from tubercular disease. I take the liberty of enclosing a copy of our last report.

The Official Replies.

To that letter I received the following reply, dated Local Government Board, Edinburgh, June 24, 1912 :—

*National Insurance Act*, 1911.

I submitted to the Local Government Board your letter of 6th instant, and in reply I am directed to reply as follows to the queries in the order they are stated therein, viz. :—

1. The term used in the National Insurance Act is tuberculosis, and it is not limited to pulmonary tuberculosis.
2. The Act covers both in-patients and out-patients.
3. Institutions approved by the Board will be able to arrange with Insurance Committees the terms on which insured persons or their dependents will be received for treatment.

I have to point out that Section 64 deals with the provision of or making grants in aid to sanatoria or other institutions for the treatment of tuberculosis, but no decision has yet been come to as to the terms and conditions on which grants will be given.

I am to add that the Board cannot yet say on what conditions their approval to sanatoria, etc., will be given, but meantime I have to refer you to Section 77 (2) and (3) of the National Insurance Act.

4. The Board can only refer you to Section 17 of the Act, and suggest that you should apply for further information to the Insurance Commissioners.—I am, etc.,

(Signed) David Brown, Assistant Secretary.

It seems from the reading of these questions that neither the Local Government Board nor the Insurance Commissioners know how the Act is to be applied in the working of it.

### The Treatment of Hospital Staffs.

The Chairman : The next point we have to consider is what arrangements hospital managers propose to make for the medical treatment of their nursing and domestic staff when the Insurance Act comes into force. Some time ago the chief hospitals here had a meeting to consider as to how they were to treat their nurses and domestic staff, and they came to the conclusion that inasmuch as they had been in the habit, when these nurses and servants turned ill, of treating them as patients in the infirmary it would hardly do to go back on that now. It would hardly do for one hospital to be turning women to the door because they were ill, and another hospital receiving them, and they were unanimously of opinion that they should continue as they had done in the past. They, therefore, thought that they might take advantage of Clause 47, which gives a reduced contribution both on the part of the employer and the employee, but before making the necessary application which is required under that clause, they went further into the matter, and they found that there would be no relaxation of the rule during those six weeks which entitled every woman to have her own medical attendant. It is, therefore, impossible in a general hospital that a nurse who happens to be unwell should have her own medical attendant coming and going about the place, and if this is to be insisted on, then I don't think we can very well avail ourselves of Clause 47, but we must go on the legal basis of the increased contribution, which means that a woman can be sent away whenever she turns ill, and leave it to the directors of the individual institution to treat each case on its own merits. I should be glad to know from Dr. M'Vail whether we are right in supposing, if we take advantage of Clause 47, to keep a nurse when treating her in the hospital, as such cases are in the habit of being treated, or whether she is still entitled to have her own medical attendant going out and in there just as it suits her, though possibly not the staff.

The Object of Clause 47.

Dr. M'Vail : I think there is a misapprehension as to the application of Clause 47. Clause 47 does not relate at all to medical benefit. It relates to sickness and disablement benefit. It is there for the purposes of meeting cases where the custom exists for the employer to provide medical attendance and treatment, and to pay wages and so forth during six weeks of illness. The object of the clause is to allow of reduced contributions by the employer where that practice exists. It is not in connection with medical benefit in the sense of altering the right of the employed person to have a free choice of doctors. If you would read the clause you would see that. There is nothing in the clause relating to medical attendance at all. It refers to the reduction of payment by the employer and the employed person where the practice exists of providing wages and housing and so forth during illness.

The Chairman : It does not alter the provision for medical attendance.

Dr. M'Vail : It has nothing to do with it.

The Chairman : It bears on the whole question, and so includes medical attendance.

The Difficulty of Medical Attendance.

Dr. M'Vail : I think the difficulty that you see could be dealt with under the first schedule of the Act. There is a provision in the first schedule that I think would meet the case. I refer to the first schedule, Part II., under (*f*), and it says " Employment in respect of which no wages or other money payment is made where the employer is the occupier of an agricultural holding and the employed person is employed thereon or where the person employed is the child of or is maintained by the employer." That is the exception and you can leave out all the rest.

Sir Robert Cranston : What has that to do with an infirmary ?

Dr. M'Vail : You are the employer of the person.

Sir Robert Cranston : It does not say so.

Mr. Barclay : Is not the condition that if no wages are paid it won't apply to nurses ?

Dr. M'Vail : No, I am talking of probationers in the first three months. With regard to nurses, if you turn to Section 15 (3) it gives you an answer where it says, " The regulations made by the Insurance Commissioners shall authorise the Insurance Committee by which medical benefit is administered to require any persons whose income exceeds a limit to be fixed by the committee, and to allow any other persons, in lieu of receiving medical benefit under such arrangements as aforesaid, to make their own arrangements for receiving medical attendance and treatment (including medicines and appliances), and in such case the committee shall, subject to regulations, contribute from the funds out of which medical benefit is payable towards the cost of medical attendance and treatment (including medicines and appliances) for such persons sums not exceeding in the aggregate the amounts which the committee would otherwise have expended in providing medical benefit for them." It is open to every nurse to make her own arrangements for medical attendance. It is not open to the hospital as a whole to make the application. It is not to the employer, but open to the individual insured person to apply to the Insurance Committee to be allowed to make her own arrangements, and every nurse can do so; and if she does that the clause takes effect.

Question: Does that apply to infectious diseases as well?

Dr. M'Vail: It applies to individuals quite independently, whether they are in a hospital or not. I am pointing out that the general application can be used by the nurse, and it has nothing to do with hospitals specially. It is to the effect that every insured person has a right to make application to the Insurance Committee to be allowed to make his or her own arrangements, both in an infectious disease hospital or other hospital.

### Medical Treatment for Hospital Employees.

Councillor W. F. Anderson (Glasgow): Might I tell you what the Corporation of Glasgow is proposing to do? First of all, we thought that because of the treatment of our nurses it would be better to exempt them altogetner or apply for exemption. We then thought that it would be a mistake, because the servants especially are much more migratory than the nurses, and, consequently, we have brought them all under the scope of the Act.

### Nurses' Choice of Doctors.

In the case of any nurse or domestic servant taken in, whether the illness lasted two weeks or six months, we gave her the very best treatment that we could get, with all the necessary medical help and a convalescent holiday if need be. She got her wages, and that was better than the Insurance scheme. What we have asked them to do is: "You have all to come under the scheme, and you have all to pay, because when you leave us it would be awkward that you should make up a different value and go into another situation," but then, instead of having half a dozen different doctors coming about any infectious hospital the nurses will have the opportunity of selecting their own medical men, and we expect that they will select what they have been in the habit of doing—the superintendent—and there would be no charge on the funds of the approved society whatever. The fee is allocated, and will be credited to the hospital; but, over and above that, if she got 7s. 6d., and that was less than she got before, the difference between them will be made good to her by the Corporation.

Dr. M'Vail: I think there must be a little misunderstanding there. If a nurse applies under Section 15 (3) to be allowed to make her own arrangements, the assumption is that she is paying an employed person's rate, and that the hospital authorities should pay the rate subject to the deduction under Section 47; and then it is not likely that the medical superintendent of the hospital will be on the panel of doctors, if a panel is formed. Not being on the panel, if the nurse wishes to be attended by the superintendent of the hospital she must apply to be allowed to make her own arrangement under Section 15 (3). The hospital superintendent will not be on the panel, because if he were on the panel any number of people might put down their names for the attendance of the same man, as it is in the infirmary for all sort of people to apply to be attended to, and the better way is for the person to take advantage of Section 15 (3).

### A Superintendent's Duties.

Councillor Anderson: That is quite contrary advice from what was given to us from Edinburgh. There is one thing—we will not have half-a-dozen medical men coming about the hospital. We were distinctly told that there would be no difficulty in arranging that. It is part of the contract and part of the superintendent's duty to attend to the staff of the hospital, and we are not going to relieve him of that part of the work. If we had known that we were in a position that we are to be, of altering the whole arrangements of the hospital, then I expect that the committee would be of opinion that they should be exempted altogether. I am satisfied that our nurses want to be insured, because they do not know how long they will be with us, but they do not want to get outside advice, and we are willing to make up the deficiency of 7s. 6d. and what they have been in the habit of getting.

### The Question of Exemptions.

Mr. D. Ross M'Kellar (Ayr): I represent the hospital in Ayr, and I ask for a little light on this word "exemption."

Mr. Alex. Duffus (Aberdeen): I think the gentleman refers to Section 51 of the Act, under which it is said that exemption may be claimed. In the case of the Aberdeen Royal Infirmary, which I represent, we noticed that clause and wrote the Commissioners or the Local Government Board to know whether exemption was possible. They would not definitely say, but the point was under consideration, and they would tell us when they had decided. With regard to the point that you refer to, sir, the question of whether the hospital should go under the full scheme or Clause 47, we in Aberdeen felt that it was on the whole difficult to understand, looking to the difficulties that there are in ascertaining exactly what this Act means and precisely what Clause 47 may mean, both in its terms and working, and probably, like Aberdonians, as we are, we felt that we should prefer to know exactly what would be the limit of our liability, and that in the meantime at all events it might be preferable to go under the full scheme till we had some experience of the working of the Act, in which case, as a sub-section of Clause 47 bears out, we can change on giving three months' notice if Clause 47 proves unworkable. We wish to fall in with other hospitals if there are good reasons given, but further in the case of these contributions the Board would wish to pay the contributions falling upon sisters and nurses. They feel that the service is the same, and that the contribution, while the Act means that it should be paid by the insured person, should be paid by the infirmary. One practical reason which might warrant such a change is this, that in the course of working we should probably find that we do not pay them enough, and that it might mean an increased salary, but it might be cheaper to agree to pay the contribution falling upon the insured and the employer rather than have to reconsider the question of salary. The same holds good to domestic servants.

### Nurses Under Clause 47.

The Chairman: Perhaps we might discuss later on the question of whether the managers are to pay both the employers' and employees' proportions; but is there anything to be said as to whether nurses should be brought under Clause 47 or not?

Sir Robert Cranston: There is one point I should like to ask a question of Dr. M'Vail, as I want to be very clear upon it. Suppose the nurses in a hospital should be under Clause 47 and they leave the infirmary or hospital, how long is it before they can get the benefits, and have they to pay back the reduced fee—that is to say, a ½d. or 1d.—have they to refund that to the Government? Is that money to be paid up before the nurses get the full benefit?

Dr. M'Vail: Do you mean that if a nurse has to pay the reduced rate owing to her employer having taken advantage of Clause 47, and if she leaves his employment and goes to some employment where Clause 47 will not apply, whether she will have to pay up the difference between leaving and going to another employment? I know of nothing in the Act that says that she will have to do so.

Sir Robert Cranston: Is there any period during which she will not get the benefit?

Mr. W. A. Tait: In Section 47, sub-section (6), there is a distinct proviso where it says, "Where such a person as aforesaid ceases to be employed within the meaning of this part of this Act, and is entitled to become a voluntary contributor paying contributions at the employed rate, paragraphs (*b*) and (*d*) of sub-section (4) shall, if he becomes a voluntary contributor, continue to apply in respect of him, and sickness benefit shall not be payable in respect of the first six weeks of any period of disease or disablement commencing after he became a voluntary contributor, but, for the purposes of calculating the rate and duration thereof, shall be deemed to have been paid during those six weeks, and, notwithstanding anything in this part of this Act, a disease or disablement shall not, for the purpose of sickness benefit, be treated as a continuation of a previous disease or disablement unless the medical practitioner attending such person certifies that it in fact is so. Providing that, if any such person at any time wishes to become an ordinary voluntary contributor, he may become such after the payment of twenty-six weekly contributions at the full rate, or, if the society which he is a member consents, after the payment of such less number of such contributions as the society may appoint."

### The Meeting's View on Contracting Out.

The Chairman: With regard to domestic servants and nurses, we seem to have come to the conclusion that we should pay the full contribution, and that any nurse or domestic servant who chooses may make use of that Clause 15 (3)—that if she wishes to make any other arrangements to contract out she can do so under 15 (3). The hospital managers have no power to compel anyone to do so, but I think that is really the mind of this meeting.

Mr. Love (Greenock): I should like to know whether each nurse needs to apply?

Dr. M'Vail: Yes, as an individual, in the same way as any other insured person. Any individual outside who, for example, does not wish to be attended by medical men on the panel, may apply to be allowed to make his own arrangements, and it does not matter whether the insured servant is an employee of any kind.

Mr. Love: Then it would not be competent to make a general application?

Dr. M'Vail: No, it must be individual. With regard to Bailie Anderson's remarks: speaking of Belvidere Hospital, I rather think he said that if they had been aware that the whole staff could be exempted, they might have taken advantage of that. The nurses are not exempted. They are still employed persons, and must conform to the Act. The point is, that it is open to every employed person to make application under Section 15 (3) with regard to medical attendance.

Councillor Anderson: But my point was that the Committee felt that the particular object of the Insurance Act was to make people better off than they were before. They have paid nothing and have got the advantage of the best advice and attention, and they have been asked for a contribution, and in return for that they are to get 7s. 6d. a week. They have got nothing and afterwards they ought to pay and they get 7s. 6d., but out of that we may say to them, "You are now getting 7s. 6d. and your medical attendance, and you may go outside. Your 7s. 6d. is to cover everything." A person is not compelled to pay 7s. 6d. a week and also keep the sick person in his house.

### Employers and Sick Persons.

Dr. M'Vail: But they do not pay 7s. 6d. a week.

Councillor Anderson: The sick person receives 7s. 6d. plus medicine and medical attendance, but the persons' employer is not supposed to keep them in the house for the period that they are paid this aliment.

Dr. M'Vail: Supposing one of your nurses in Belvidere was suffering from an infectious disease, I am afraid that under the Public Health Act you would be bound to keep her.

Councillor Anderson: But as a matter of fact, they do not suffer from infectious diseases as much as other diseases, and up to the present they have got everything and got their medical attendance and convalescent period paid. Now they feel that they are asked to pay 3d. a week, and they are asked to select a doctor that they do not want. They have got to select a doctor out of a panel, and the superintendent has been in the habit of treating them.

Dr. M'Vail: I am awfully sorry if I have not made it clear that they do not require to do anything of the kind—that they can apply for permission to make their own arrangements.

Sir Robert Cranston: Supposing that is not granted?

Dr. M'Vail: That lies with the Commissioners.

Sir Robert Cranston: But you say that they are to apply, and an application does not mean a grant.

Dr. M'Vail: No.

Mr. Barclay: Under Section 47 if a nurse has been in a hospital under Clause 47 and pays her contribution of 2d. or 2½d. and leaves that hospital and goes to another hospital not under Clause 47, is there a certain period in which she gets no sickness benefit in the new hospital?

### Hospitals Not Under Clause 47.

Dr. M'Vail: You mean, would she not get six weeks' medical attendance in the new institution? I am not a lawyer, but I would assume that if she goes into an institution which is not under Clause 47 she would have the right of medical attendance and medicine under the Act.

Mr. Barclay: I understood that there were six weeks in which she got no medical benefit.

Dr. M'Vail: If there is any doubt, will you write a question, and I shall see that it is answered?

Mr. I. Nelson Gray: Is it the individual that comes under Clause 47, or is it every institution?

Dr. M'Vail: Under Clause 47 it is the employer who makes application for being allowed to pay the reduced amount of money weekly by himself, and his employee to be allowed to pay an amount weekly. That is totally different from Clause 15 (3), where the individual insured person may apply to be allowed to make her own arrangements. They are totally different subjects.

### The Second Resolution.

The Chairman: Perhaps it may be as well to have a general resolution on this subject, and I have to put this to the meeting—"In the opinion of this Conference, the hospitals should not make any application for a special order under Section 47, but should place their nursing and domestic staffs under full scale of payment."

This was duly seconded.

Mr. Duffus (Aberdeen) : Would it not be advisable to put "in the meantime," which shows that you are not finally shutting the door?

The Chairman : I adopt that. Do you agree?

Councillor Anderson : But I do not want our nurses to be in a worse position than before. That is the attitude of our Corporation.

Sir Robert Cranston : I think that is a misapprehension. If we take the reduced payment we will place our nurses in a different position.

### Poor-Law and Municipal Hospitals.

Councillor Macpherson (Edinburgh) : I am not here representing a voluntary hospital, but rather with a watching brief in the interests of our nurses and domestic staff in our city hospital in Edinburgh. We have received the Local Government Board circular, and have replied to it as to what we are prepared to do when the Insurance Act comes into force. If our offer which we have submitted to the Local Government Board be accepted, I presume that we will then become a voluntary hospital under the Insurance Act. I do not associate myself with Bailie Anderson in this protest.

The Chairman : It won't prevent the Glasgow Corporation from doing anything they like.

Councillor Anderson : All right.

The Chairman : The only thing is that, seeing the great difficulty in the way of working Clause 47, we should in the meantime pay the full contributions.

Councillor Anderson : That is what we have agreed, plus what we care to give them.

Mr. Love (Greenock) : Does it include the matron and house surgeons?

The Chairman : No, we are only dealing with the nursing and domestic staffs at present.

Then the resolution is carried unanimously.

### Should Hospitals Pay Employees' Contributions?

The Chairman : There is a great deal to be said on both sides, but my own feeling is that it is a mistake to pay the employees' contributions. (Hear, hear.) Really, the better way would be to raise the salaries, and I think it would be a great mistake to pay employees' contributions in anything of this kind. I do not say that the nurses are the people to whom this should apply, but the fact that a Bill such as this costs money requires to be brought home. I do not know whether it is necessary to move any resolution on that subject, but that is my private opinion. (Cries of "Agreed.")

Councillor Macpherson (Edinburgh) : There is a letter that has been before our Health Committee in Edinburgh, and I mention it merely in an informal way. A number of gentlemen thought that although we know that at the present time we require to come under the Insurance Act and accept the whole terms of it, in the case of the Royal Infirmary and such institutions where the staff is very likely to be continued on former conditions, it was felt that although the nurses or domestics may be ill, they will continue to receive full wages and medical attendance. That is generally the indication of the public mind as far as I am concerned at the present moment. There is a feeling amongst all of us that by and by when the Act is under operation the Insurance Commissioners should be asked to take into consideration the fact that large institutions would be contributing a large fund for other parties to get the benefit of. We were of opinion that if we continued to pay during illness the Commissioners should be asked to consider the contributions made particularly by the employer, and that large institutions with many hundreds of nurses being dealt with in this sympathetic way ought not to pay the full complement required by the Act. I merely mention this in order that others may take it into consideration and lead to some joint action, and then the case could be submitted to the Insurance Commissioners.

The Chairman : That comes on later.

### Unsalaried Probationers.

Dr. Mackintosh : There is one point on which I would like to have the opinion of Dr. M'Vail, it has reference to probationers who receive no salary from a hospital during the first three months of their service. What position is an hospital in with regard to probationers who receive no salary? Does the hospital pay both the contributions of the employer and the employee until these probationers receive salary? If Dr. M'Vail would kindly answer the question in writing I would circulate his reply to the members of the British Hospitals Association for their guidance.

Dr. M'Vail : I think this is the answer—Is it not found in the fact that to-day I called Sir Robert Cranston's attention to the fact that in the second part of First Schedule under Clause 47 it says "Employment in receipt of which no wages or other money payment is made where the employed person is maintained by the employer." That is excepted. It does not refer merely to agricultural labourers.

Dr. Mackintosh : It is a matter of very great importance to those of us who have to administer hospitals. I have asked the same question on one or two occasions and have been informed that unless the engagement is a "contract of service" the institution has to pay the employee's contribution as well as its own.

Dr. M'Vail : I can only read to you what the Act says. The first schedule of the Act is in two parts—first of all, employment in regard to health insurance and certain exceptions and things that are not under the Act, and one of these is "Employment in receipt of which no wages or other money payment is made where the person employed is maintained by the employer."

Sir Robert Cranston : Is he or she exempted altogether from payment?

Dr. M'Vail : Yes.

Sir Robert Cranston : Both employer and employee.

Dr. M'Vail : As I take it. You are training your probationer and she is getting her board and wages, and she is being maintained by the employer. It is a lawyer's question and I am not a lawyer.

Sir Robert Cranston : Neither am I, but with all due respect you have not answered my question. I admit that what you say is perfectly true, but have the employers to pay?

Dr. M'Vail : No.

Sir Robert Cranston : They are both exempted?

Dr. M'Vail : I am not speaking as a lawyer, but it appears to me that the exception is perfectly clear, and it applies both to the employer and the employee.

Sir Robert Cranston : Then I am quite satisfied.

The meeting concluded with cordial votes of thanks to Dr. M'Vail and the Chairman.

